# Comparison of the Efficacy between Conventional Moxibustion and Smoke-Free Moxibustion on Knee Osteoarthritis: A Randomized Controlled Trial

**DOI:** 10.1155/2019/1291947

**Published:** 2019-03-14

**Authors:** Ling Luo, Mo Liao, Jia-xi Peng, Qi Ma, Jian-ying Zhou, Lin-lin Zhu, Xiang Wang, Sha-Sha Chen, Hai-Yan Yin, Qiao-Feng Wu, Cheng-shun Zhang, Peng Lv, Yong Tang, Shu-guang Yu

**Affiliations:** ^1^School of Acupuncture and Tuina, Chengdu University of Traditional Chinese Medicine, Chengdu, Sichuan, China; ^2^Suining Municipal Hospital of Traditional Chinese Medicine, Suining, Sichuan, China; ^3^College of Medicine, Chengdu University, Chengdu, Sichuan, China; ^4^Key Laboratory of Sichuan Province for Acupuncture & Chronobiology, Sichuan, China

## Abstract

**Objective:**

The aim of this study was to investigate the difference of efficacy between conventional moxibustion (CM) and smoke-free moxibustion (SM) for patients with osteoarthritis of the knee (KOA).

**Methods:**

This is a multicentre, randomized, single blinded, parallel-group clinical trial. Patients with KOA were randomly allocated to CM group (69) and SM group (69) in 7 hospitals of China. Moxibustion treatment in 12 sessions over 4 weeks was administrated at 3 acupuncture points (EX-LE4, ST35, and ST36). Patients completed standard questionnaires at baseline and after 2 weeks, 4 weeks, 8 weeks, and 12 weeks. The primary outcome was the Western Ontario and McMaster Universities Osteoarthritis Index (WOMAC) from the baseline to 4 weeks. The secondary outcomes include Visual Analogue Scale (VAS) and Patient Global Assessment score (PGA).

**Results:**

Analyses showed that the WOMAC score improved in pain (95% CI,-0.1[-1.2 to 0.9], p=0.76), stiffness (95% CI,-0.1 [-0.5 to 0.3], p=0.71), and function (95% CI, 2.2 [-1.3 to 5.8], p=0.22) compared between the two groups at 4 weeks, as well as the VAS score (95% CI,0.1 [-0.3 to 0.6], p=0.60). Similar results presented at 8 and 12 weeks. No statistically significant difference was observed between CM and SM groups for outcome measurements.

**Conclusions:**

It suggested that smoke generated during moxibustion treatment does not affect the efficacy of moxibustion in the treatment of KOA, which should be taken into account to be removed for the sake of reducing environmental pollution or moxa smoke exposure of acupuncturists or patients. This trial is registered with Clinical Trials.gov, NCT02772055.

## 1. Introduction

Knee osteoarthritis (KOA) is the most common form of arthritis and associated with significant pain and declines in physical function [1]. It is typically a cause of disability, limitation of activity, especially in elderly patients (more common in women than in men) older than 50 Years [2–4]. Anti-inflammatory drugs used to treat the symptoms of arthritis are associated with various adverse events [5]. Some recent international guidelines advocating nonpharmacological interventions are central to improve pain, function, and quality of life [6–9]. Moxibustion as a representative nondrug intervention in traditional Chinese medicine (TCM) is frequently used in patients with arthritis and joint pain [10].

Acupuncture is the most popular of alternative medical systems, there is some evidence that it can be effective in treating pain and dysfunction in patients with KOA [11–13]. Moxibustion is a traditional Chinese method of acupuncture treatment, in which the heat generated by burning herbal preparations (Mugwort or Moxa) is applied to stimulate acupuncture points [14]. Recent studies have suggested that moxibustion has anti-inflammatory or immunomodulatory effects against chronic inflammatory conditions, improved blood circulation, and released chemicals that can alleviate pain in humans [15, 16]. Moxibustion has been confirmed sufficiently to demonstrate that it does have benefits for KOA in many studies including clinical trials and systematic review [17–22]. This clinical trials and review suggested that moxibustion could be beneficial for pain control in patients with KOA. The mechanism of moxibustion is mainly related to the thermal and radiation effects, pharmacological activity of moxa, and its combustion products (volatile oil, brown tar-like substances, and moxa smoke) [23]. Moxa smoke, as a primary combustion product of the moxibustion, with its potential effect on the health and environment may be controversial [23, 24]. A recent evidence summary of moxa smoke has antibacterial and antiviral effects to treat various conditions including wound infections, vaginal itching, uterine prolapse, anal fistula, and common warts [25–27]. However, it was reported that high levels of monoaromatic hydrocarbons, formaldehyde, and polycyclic aromatic hydrocarbons in moxibustion treatment room may cause adverse effects on human health [28–30]. Furthermore, people gradually increased environmental awareness and the safety of the smoke produced by burning moxa [31–33].

Given the improvement of environment awareness and safety of smoke, acupuncturists prefer to use smoke purification device to remove moxa smoke during the moxibustion treatment. However, having questions about the difference of clinical efficacy between conventional moxibustion (CM) and smoke-free moxibustion (SM), recommended treatments for this population remain unanswered. Therefore, we carried out a randomized controlled clinical trial to assess the clinical efficacy of CM and SM for patients with KOA.

## 2. Methods

### 2.1. Setting and Study Population

We performed a multicentre, randomized, single blinded, parallel-group design clinical trial for 13 weeks from June, 2016, to May, 2017. The trial protocol adhered to the CONSORT (Consolidated Standards of Reporting Trails) and STRICTA (Standards for Reporting Interventions in Clinical Trials of Acupuncture) guidelines [34] and has been published [23]. The institutional human ethics committee approved the study. All participants gave their oral commitment and written informed consent.

Participants were recruited through advertisements in local newspapers and the hospital website, posters in local communities and the KOA outpatient and inpatient clinics at 7 clinical research centers in China will participate in this trial: Central Hospital of ZiBo, Chengdu First People's Hospital, Sichuan Second Hospital of TCM, Pi County People's Hospital, Xinjin County TCM Hospital, Qionglai TCM Hospital, and Nanjing Hospital of TCM. The inclusion criteria were as follows: aged between 40 and 75 years, diagnosed with KOA formulated by the American College of Rheumatology (ACR), average severity of knee pain no more than 7 points on a visual analogue scale for most days during the past month, agreed with no paregoric usage during the whole treatment phase, and willingness to participate in a randomized study and to sign the informed consent form. Participants will be excluded if they have any of the following conditions: pain in the knee that may be caused by inflammatory, malignant, or autoimmune disease or by traumatic injury; serious diseases including cancer, uncontrolled hypertension, diabetes mellitus requiring insulin injection, life-threatening cardiovascular or neurological events, chronic respiratory disease, a hemorrhagic disorder, or serious mental diseases; knee replacement surgery, arthroscopy of the affected knee within the past year, steroid or hyaluronic acid injection in the knee joints within the previous 3 months; life-threatening cardiovascular or neurological events within the past year; physiotherapy or other treatments for osteoarthritis knee pain (with the exception of nonsteroidal anti-inflammatory drugs) during the previous 4 weeks; current participation in another clinical trial; accepted acupuncture, moxibustion, cupping, or herbal medicine within the past 4 weeks.

### 2.2. Interventions

We used traditional Chinese medicine style of moxibustion. All project general practitioners (GPs) had completed at least 5 years of training in acupuncture and moxibustion, registered as Chinese medicine practitioner by the National Health and Family Planning Commission of the People's Republic of China [23]. Project GPs were trained for one day by an investigator who is an experienced medical acupuncturist, to standardise all aspects of the treatment protocol. Two separated moxibustion rooms were provided for CM and SM group by the centers, with opening doors and windows in the process of treatment. Selection of acupoints is based on TCM meridian theory to treat knee joint pain, known as the syndrome and some similar studies [13, 17, 18, 35, 36]. All patients received moxibustion on 3 obligatory points, including ExLE4, ST35, and ST36 [17, 23, 37]. Thirty-minute treatments were delivered 3 times per week, for 4 weeks, with 12 sessions in total permitted.

Two groups will use a moxa device (Yijiu moxa device, Maanshan, Anhui, China) at acupoints. The moxa pillar is cylindrical, 1.5cm in diameter, and 3cm in length; a lighted moxa pillar will be put into the moxa device attached at each acupoint making sure the patients feel warm but not scorching [23]. Compared with CM group, a device designed specifically (Shenzhen Conyson Electronic Technology Co., Ltd., C200 moxa smoke purification device, Shenzhen, China) was applied to remove the moxa smoke for the SM group in the process of moxibustion; the selection of acupoints and other intervention were same in both groups.

### 2.3. Randomization and Blinding

After baseline measurements, the participants were randomized to SM and CM group. Central randomization, the randomization sequence, was prepared by (SPSS 16.0, SPSS Inc., Chicago, IL, USA) a biostatistician with no clinical involvement in the trial. Randomization sequence was accessed in a password-protected computer file and allocated to each clinical research center by investigator.

The participants in the SM group and CM group were blinded; the patients were separated and treated in two different rooms and unaware of the assignments. Project GPs could not be blinded. Meanwhile, this procedure ensured that participants, outcomes assessors, data collectors, and statisticians were blinded to treatment allocations throughout the trial.

### 2.4. Measurements

Participants completed questionnaires at baseline, at the 2- and 4-week visits (the latter was the end of treatment) and at the 8- and 12-week follow-up visits (by telephone). The primary outcome was reliable reported pain, stiffness, and function measures for osteoarthritis by the global scale value of the Western Ontario and McMaster Universities Osteoarthritis Index (WOMAC) questionnaire [36], and higher scores indicate more severe impairment. Secondary outcomes included average knee pain, using the 100mm Visual Analogue Scale (VAS) questionnaire (score range, 0-10), higher scores indicating worse knee pain; the patient global assessment (PGA) score was used to evaluate overall improvement by a 5-point ordinal scale (much improved, minimally improved, no change, minimally worse, or much worse) after treatment. Side effects of treatment, adverse events, and use of medication were also recorded. All data acquisition, processing, and analyses were performed by study staff who were blinded to group allocation.

### 2.5. Sample Size

We designed our trial to investigate the efficacy between CM and SM for patients with osteoarthritis of the knee (KOA). The significance level is 5% and statistical power is 90%, consistent with our pilot study and a previous trial on moxibustion for KOA [37]. With an estimated loss-to-follow-up rate of 15%, we planned to enroll 138 participants in the 2 groups, with 69 participants in each group from 7 centers in China.

### 2.6. Statistical Analysis

The baseline characteristics and clinical outcomes described are based on the intention-to-treat (ITT) population. Analysis was performed with the SPSS software (SPSS 21.0, SPSS Inc, Chicago, IL, USA). Estimates of the treatment effects for outcomes data are presented as difference in mean change, with 95% CI. *P* values less than 0.05 were considered statistically significant and tests were 2-sided. Demographic and baseline data will be analyzed with standard, descriptive statistics. We performed the WOMAC subscale and VAS score comparisons between CM and SM group, as well as pairwise comparisons between the 2 groups with baseline. Differences in mean changes for each outcome at each time were compared using nonparametric tests (*t* test). Global changes were compared between groups using the *χ*2 test. All statistical analyses were performed on blinded group allocations.

## 3. Results

**Figure 1:**
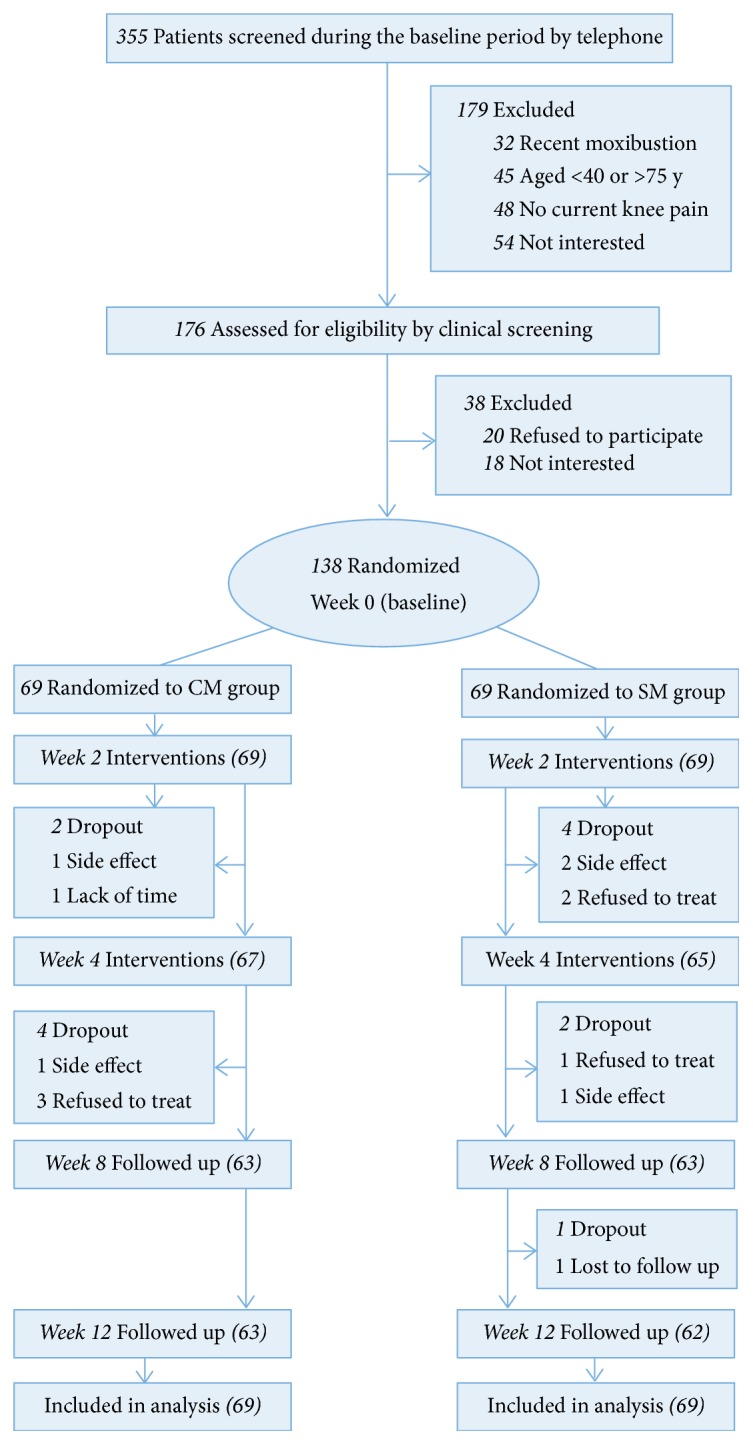
Flow of participants through the trial.

Of 355 patients screened by telephone, 176 attended the clinical screening. Thus, 138 were randomly assigned to treatment (Figure 1). After 12 weeks, 13 participants (6 in the CM group and 7 in the SM group) had dropped out and were not contactable. Participants had a mean age of 59.9 years and 107 (76%) were women.  Table 1 presents the baseline characteristics of the patients with KOA. Percentage of 58 participants had been diagnosed with KOA for 1 to <5 years. The groups were balanced at baseline (Table 1).

Tables 2 and 3 summarize continuous outcomes. There were no significant differences in primary outcomes and secondary outcomes between CM and SM groups at 4 weeks (Tables 2 and 3,* P*> 0.05). Both conventional moxibustion (CM) and smoke-free moxibustion (SM) resulted in modest improvements in pain, stiffness, and function compared with baseline after treatment at 4 weeks, and the effect was maintained at 12 weeks (Tables 2 and 3). We found no statistically significant group differences in the primary outcomes and secondary outcomes at any time point.

### 3.1. Adverse Events

Seven patients (2 in the CM group and 5 in the SM group) reported adverse events during the 12 weeks. Six patients (2 in the CM group and 4 in the SM group) complained of a burning sensation after moxibustion in the acupoints located on the knee. One described blister of the knee after moxibustion was removed from the acupoints. All adverse events were reported as mild or moderate, and none required special medical interventions. The 4 patients recovered from the adverse events and did not withdraw from the trial, and three patients (1 in the CM group and 2 in the SM group) were missing data from the adverse events.

## 4. Discussion

This study was designed to evaluate the clinical efficacy of CM and SM for treating KOA. In this study, patients with KOA who received moxibustion (including CM and SM) had less pain and better function after 4 weeks than baseline; after 4 and 12 weeks, exploratory analysis indicated that the differences between CM and SM were no longer significant. Although considerable improvements were observed at each time, no differences between the CM and SM groups were found.

Our findings agree with systematic review and individual patient data meta-analysis on moxibustion efficacy for KOA [17–22]. To our knowledge, our study is the first reported randomized controlled clinical trial to assess the clinical efficacy of CM and SM for patients with KOA. Recent research has focused on the composition and the safety of moxa smoke. However, few studies were concerned about the effect of moxibustion with removal of the moxa smoke [30, 38].

To consider the safety of moxa smoke, it is important to assess the types and concentrations of potentially harmful substances generated. PM exposure has used a variety of metrics for PM, including total suspended particles (TSP), PM10 and PM2.5 (PM with an aerodynamic diameter of < 10 *μ*m and < 2.5 *μ*m, respectively) [39]. In our study, an air cleaner (C200, moxa smoke purification device, Shenzhen, China) was selected to be placed at the top of the moxibustion device to remove the moxa smoke during thirty minutes to mimic clinical practice; meanwhile, airborne PMs of moxa smoke were measured using ZR-3920 (ambient airborne particulate sampler, Lunray, Qingdao, China) nearby the moxibustion device. The mean concentration of TSP, PM10, and PM2.5 was 13 ug/m^3^, 15.6 ug/m^3^, and 12 ug/m^3^, respectively. However, lacking evidence on the moxibustion rooms of PM exposure in this level range that may determine toxicity, a general mass-based standard was promulgated. In our trial, a novel moxibustion device which is applied to remove the moxa smoke, conducting a pilot study to evaluate moxibustion effectiveness in a clinical setting, was successful. The device has been used in clinical practice to remove the moxa smoke, and our design is based on clinical application. Our results show that moxa smoke does not affect the efficacy of moxibustion in the treatment of KOA. The improvement of environment awareness and safety of smoke in moxibustion treatment room may have adverse effects on patients and medical personnel's health [29, 30]. Therefore, our findings should be considered in therapeutic decision making during the moxibustion treatment in patients with KOA.

Although moxibustion has a positive effect on KOA, those factors promoting moxibustion to produce treatment effects also are primary contributors to various adverse events, such as burn wounds, pruritus, fatigue, blisters, and skin flushing in moxibustion [17, 19, 37, 40]. In our trials, seven patients reported possible adverse events during the 12 weeks. Six patients complained of a burning sensation and 1 described blisters of the knee after moxibustion was removed from the acupoints; all adverse events were reported as mild or moderate. It must be noted that all adverse events can cure without medical care [17] and most patients regarded moxibustion as a safe complementary and alternative medicine [19]. It was reported that patients receiving moxibustion did not experience adverse events [41]; several large surveys have also provided evidence that moxibustion is a relatively safe treatment [42–44]. The possible occurrence of these adverse events during moxibustion treatment should be monitored with caution.

One could argue that all of the included studies had a high risk of bias. Our trial had high internal validity, shown by adequate recruitment, central randomization, high follow-up rates, and effective blinding of the research team. The protocol was published previously and experienced practitioners delivered the interventions in accordance with standard study. The nature of the moxibustion procedure makes blinding difficult, as patients might expect warmth to radiate from the burning moxa. In our trial, blinding was successful in part because all of the patients received the moxibustion treatment and 2 separated moxibustion rooms were provided for 2 groups by the centers; the only difference was the moxa smoke purification device (C200) applied for the SM group in the process of moxibustion. Therefore, participants were unable to communicate with each other about their treatment experiences. The data from questionnaire responses indicate that neither psychological factors such as previous experience nor study procedures such as moxa heat sensation had a significant effect, suggesting that the treatment and control procedures were equally credible. Due to the difficulty of double-blind methodology in acupuncture and moxibustion, it was not possible to blind therapists to treatment, and it was not necessary to set up the wait-list group [23].

In conclusion, moxa smoke does not affect the efficacy of moxibustion in the treatment of knee osteoarthritis. Our findings likely only apply to patients with clinically diagnosed KOA and moderate pain or function. In addition, it is essential to consider the safety of moxa and medical personnel's occupational exposure after moxibustion treatment.

## Figures and Tables

**Table 1:** Baseline characteristics by study group.

Characteristic	CM	SM	All Participants
	(n=69)	(n=70)	(n=139)
*Demographic Characteristic*			
Female sex, No. (%)	54(78)	53(77)	107(76)
Age, mean (SD), y	60.7(9.0)	58.2(8.0)	59.9(8.4)
Height, mean (SD), cm	159.9(6.6)	160.1(7.5)	159.7(6.9)
Weight, mean (SD), kg	61.3(14.2)	63.7(10.3)	62.7(11.0)
BMI, mean (SD)	24.9(3.6)	24.2(3.7)	24.5(3.6)

*Symptom duration, No. (*%)			
<1 y	2(3)	3(4)	5(4)
1 to <5 y	44(64)	36(53)	80(58)
5 to<10 y	13(19)	16(23)	29(21)
≧10 y	10(14)	14(20)	24(17)

*Medication use, No. (*%)			
Glucosamine products	7(10)	7(10)	14(10)
Analgesia	3(4)	1(1)	4(3)

*Past treatment, No. (*%)			
Moxibustion	3(4)	1(1)	4(3)
Acupuncture	0	1(1)	1(1)
Injections	1(1)	0	1(1)

Abbreviations: SM, Smoke-Free Moxibustion; CM, Conventional Moxibustion; BMI, Body Mass Index (calculated as weight in kilograms divided by height in meters squared).

**Table 2:** Outcome measurements during the entire study.

Outcome Measure	CM(n=69)	SM(n=69)	Mean Difference	*P *Value
			(95% CI)	
*Baseline*				

*WOMAC subscale score *				
Pain	7.4(3.4)	7.6(2.9)	-0.2(-1.3 to1)	0.81
Stiffness	1.6(1.6)	1.6(1.6)	0(-0.6 to 0.6)	0.98
Function	25.2(11.8)	22.9(12.7)	2.3(-2.0 to 6.7)	0.29

*VAS score*	4.1(1.4)	4.0(1.4)	0.1(-0.4 to 0.6)	0.62

*2 week*				

*WOMAC subscale score *				
Pain	5.8(3.7)	6.2(3.1)	-0.4(-1.6 to 0.8)	0.53
Stiffness	1.2(1.5)	1.3(1.4)	-0.1(-0.5 to 0.5)	0.94
Function	18.9(11.3)	18.0(11.1)	0.9(-3.0 to 5.0)	0.63

*VAS score*	3.0(1.4)	3.1(1.5)	-0.1(-0.6 to 0.4 )	0.61

*4 week *				

*WOMAC subscale score *				
Pain	4.1(3.0)	4.2(2.9)	-0.1(-1.2 to 0.9)	0.76
Stiffness	0.7(1.2)	0.8(1.1)	-0.1(-0.5 to 0.3 )	0.71
Function	15.1(10.8)	12.9(9.2)	2.2(-1.3 to 5.8)	0.22

*VAS score*	2.2(1.3)	2.1(1.3)	0.1(-0.3 to 0.6)	0.60

*8 week*				

*WOMAC subscale score *				
Pain	4.1(3.1)	4.2(3.0)	-0.1(-1.2 to 0.1)	0.81
Stiffness	0.7(1.2)	0.6(1.0)	0.1(-0.3 to 0.4)	0.79
Function	15.1(10.8)	12.7(8.9)	2.4(-1.1 to 5.9)	0.18

*VAS score*	2.2(1.2)	2.1(1.2)	0.1(-0.3 to 0.6)	0.62

*12 week*				

*WOMAC subscale score *				
Pain	4.1(3.2)	4.0(2.8)	0.1(-1.0 to 1.2)	0.86
Stiffness	0.7(1.3)	0.6(1.0)	0.1(-0.3 to 0.5)	0.73
Function	15.6(10.6)	12.4(9.3)	3.2(-0.2 to 6.9)	0.06

*VAS score*	2.1(1.2)	2.1(1.2)	0(-0.4 to 0.5)	0.83

Abbreviations: SM, Smoke-Free Moxibustion; CM, Conventional Moxibustion; WOMAC, Western Ontario and McMaster Universities Osteoarthritis Index; VAS, Visual Analog Scale.

CM and SM group data are expressed as mean (SD) unless stated otherwise.

**Table 3:** PGA at follow-up, values are no. (%) of participants unless stated otherwise.

Outcome Measure	CM(n=69)	SM(n=69)	*P *Value
*2 week*			
Much improved	12(18)	5(7)	0.14
Minimally improved	41(59)	50(72)	
No change	14(20)	10(15)	
Minimally worse	0	0	
Much worse	0	0	

Missing data	2(3)	4(6)	/

*4 week*			
Much improved	21(30)	24(35)	0.33
Minimally improved	36(52)	37(53)	
No change	6(9)	2(3)	
Minimally worse	0	0	
Much worse	0	0	

Missing data	4(6)	2(3)	/

*8 week*			
Much improved	19(27)	18(26)	0.36
Minimally improved	40(58)	43(62)	
No change	4(6)	1(2)	
Minimally worse	0	0	
Much worse	0	0	

Missing data	6(9)	1(2)	/

*12 week*			
Much improved	21(30)	17(24)	0.27
Minimally improved	38(55)	44(64)	
No change	4(6)	1(2)	
Minimally worse	0	0	
Much worse	0	0	

Missing data	6(9)	7(10)	/

Abbreviations: PGA, patient global assessment; SM, smoke-free moxibustion; CM, conventional moxibustion.

## Data Availability

The data used to support the findings of this study are available from the corresponding author upon request.
